# Supplementary material for: Feather moult and bird appearance are correlated with global warming over the last 200 years
Source: Nat Commun. 2019 Jun 10;10:2540. doi: 10.1038/s41467-019-10452-1 (PMC6557852; doi:10.1038/s41467-019-10452-1)
Supplement: Supplementary file 1 — Supplementary Information [file 41467_2019_10452_MOESM1_ESM.pdf]

Feather moult and bird appearance are correlated with global warming over the last 200 years

Kiat et al.

SUPPLEMENTARY INFORMATION

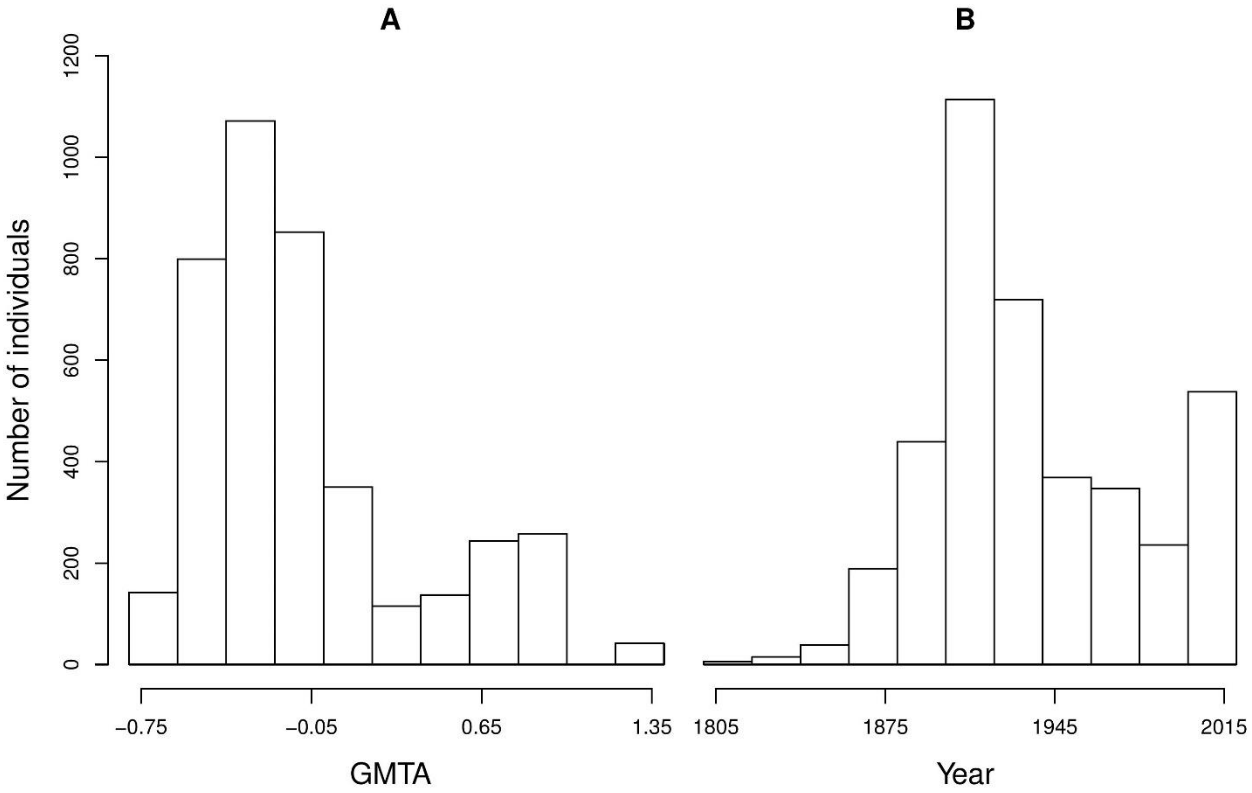

Supplementary Figure 1. Histograms of the examined individuals (*n* = 4012) in relation to (A) Global Mean Temperature Anomalies (GMTA), and (B) year.

A

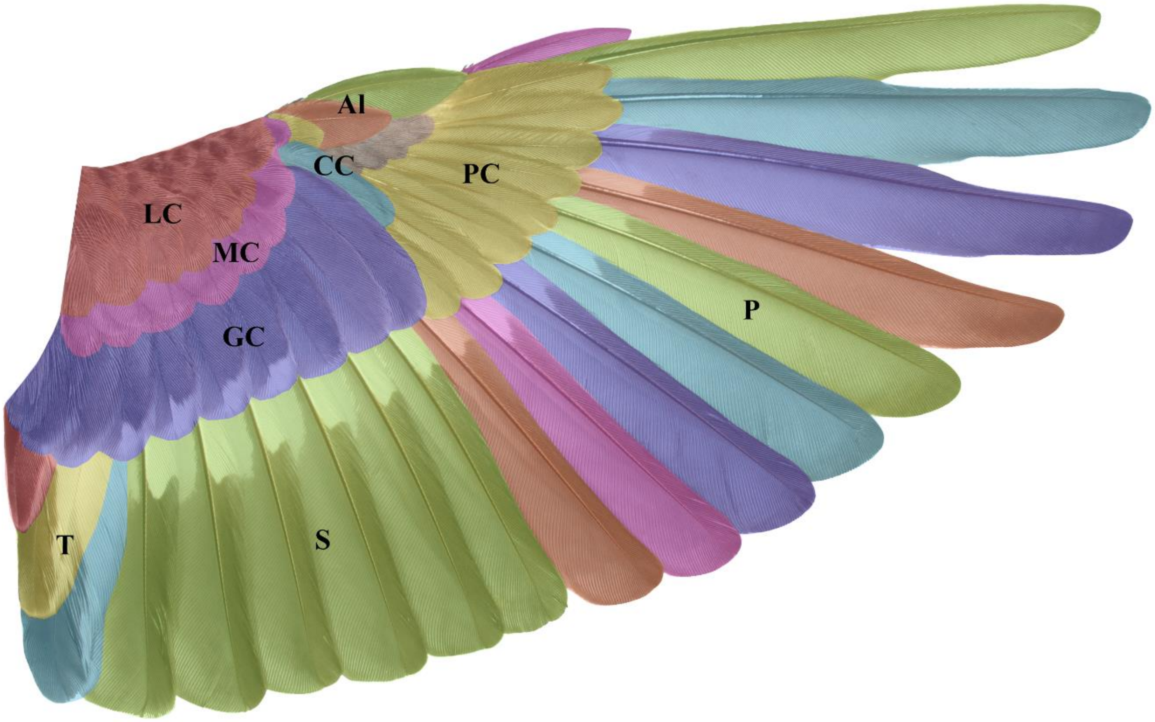

B

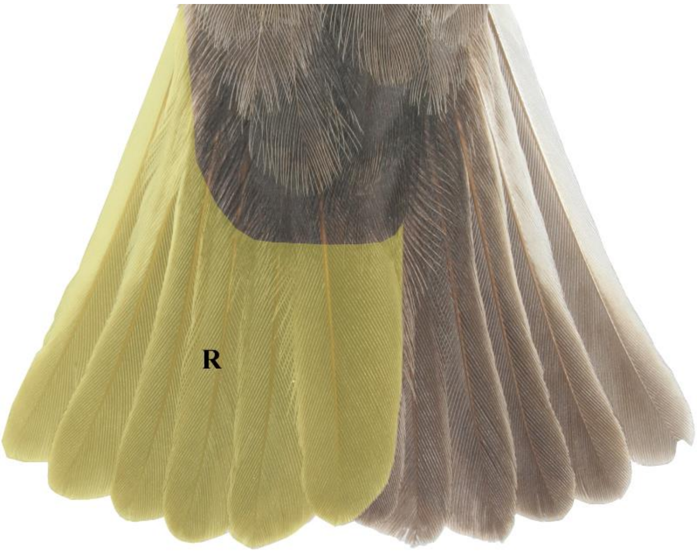

Supplementary Figure 2. Wing and tail feather tracts that were tested in this study and their abbreviations: (**A**) Illustration of the wing and (**B**) Illustration of tail (both of *Ficedula albicollis*).

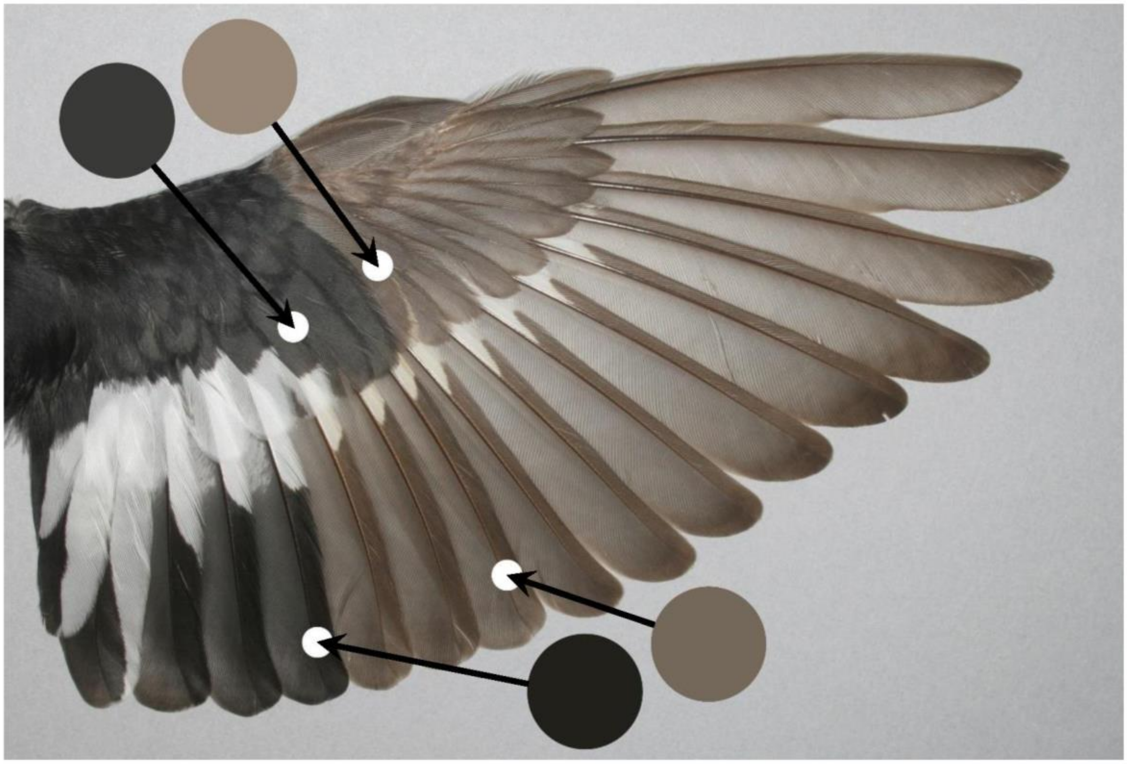

Supplementary Figure 3. Collared Flycatcher *Ficedula albicollis* male that was photographed after his post-juvenile moult (April 2016). The circles display the colour difference between moulted and non-moulted feathers in the greater-coverts and the secondaries, which were sampled using Photoshop version 6.0.1.a. The colour difference between moulted and non-moulted feathers is prominent.

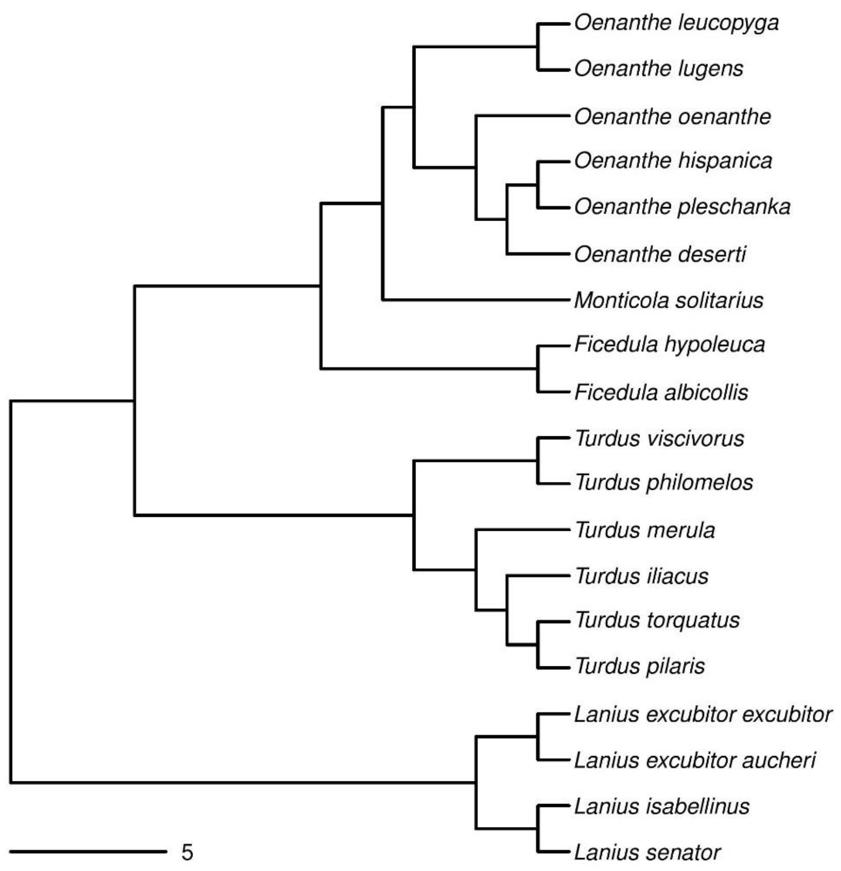

Supplementary Figure 4. The phylogenetic tree of the 19 examined species that was used in the Phylogenetic Generalized Least Square (PGLS) analysis (see Supplementary Table 5). The tree is based on an analysis of global bird diversity, using 10,000 trees obtained from BirdTree.org. The consensus tree was built using BEAST version 1.8.4. The scale (bottom left) represents 5 million years.

Scientific name	Common name	Subspecies included	Molt strategy	Migration distance (km)	Sexual-dichromtism after the moult	Sample-size	Data period
Oenanthe oenanthe	Northern Wheatear	*oenanthe* , *libanotica*	pre-migration	7200	No	224	1849-2016
Oenanthe deserti	Desert Wheatear	*deserti* , *homochroa*	pre-migration	1100	Yes	145	1834-2016
Oenanthe hispanica	Black-eared Wheatear	*hispanica* , *melanoleuca*	pre-migration	2700	Yes	231	1812-2016
Oenanthe pleschanka	Pied Wheatear	Monotypic	pre-migration	4400	Yes	195	1864-2016
Oenanthe lugens	Mourning Wheatear	*lugens* , *persica*	pre-migration	0	No	102	1822-2015
Oenanthe leucopyga	White-crowned Wheatear	*leucopyga* , *ernesti*	pre-migration	0	No	101	1825-2016
Monticola solitarius	Blue Rock-Thrush	*solitarius* , *longirostris*	pre-migration	1300	Yes	107	1812-2015
Turdus philomelos	Song Thrush	*philomelos* , *hebridensis* , *clarkei* , *nataliae*	pre-migration	2000	No	422	1855-2015
Turdus iliacus	Redwing	*iliacus* , *coburni*	pre-migration	2700	No	314	1805-2016
Turdus viscivorus	Mistle Thrush	*viscivorus* , *deichleri*	pre-migration	1500	No	164	1846-2016
Turdus pilaris	Fieldfare	Monotypic	pre-migration	1700	No	317	1805-2015
Turdus merula	Blackbird	*merula* , *azorensis* , *cabrerae* , *mauritanicus* , *aterrimus* , *syriacus* , *intermedius*	pre-migration	300	Yes	491	1826-2016
Turdus torquatus	Ring Ouzel	*torquatus* , *alpestris* , *amicorum*	pre-migration	2500	Yes	239	1843-2016
Ficedula hypoleuca	Pied Flycatcher	*hypoleuca* , *tomensis* , *iberiae* , *speculigera*	post-migration	5200	Yes	182	1809-2015
Ficedula albicollis	Collared Flycatcher	Monotypic	post-migration	6900	Yes	112	1834-2016
Lanius isabellinus	Isabelline Shrike	*isabellinus* , *phoenicuroides*	post-migration	3400	Yes	105	1824-2016
Lanius senator	Woodchat Shrike	*niloticus* , *senator* , *badius*	post-migration	3400	Yes	184	1809-2015
Lanius e. excubitor	Great Grey Shrike	*excubitor* , *homeyeri*	pre-migration	900	No	228	1861-2016
Lanius e. aucheri	Southern Grey Shrike	*aucheri* , *elegans* , *algeriensis*	pre-migration	0	No	149	1821-2016

Supplementary Table 1. List of the study species including information on their moult strategy, migration distance, sexual-dichromatism, sample-size and data period (years).

Scientific name	Data source										
	Natural History Museum (Tring; UK)	Museum National d'Histoire Naturelle (Paris, France)	National History Museum of Denmark (Copenhagen, Denmark)	Museum für Naturkunde (Berlin, Germany)	Museo Nacional de Ciencias Naturales (Madrid, Spain)	Naturhistoriska Riksmuseet (Stockholm, Sweden)	Natural History Museum Vienna (Vienna, Austria)	Steinhardt Museum of Natural History (TAU) and National Natural History Collections (HUJ) (Israel)	Naturhistorisches Museum (Basel, Switzerland)	Pictures obtained from the internet	Field
Oenanthe oenanthe	91		50			35	17	24		1	6
Oenanthe deserti	51	19	17			9	21	7		10	11
Oenanthe hispanica	128	7					57	16		11	12
Oenanthe pleschanka	126		16	12		7	7			27	
Oenanthe lugens	40	8		7			3	29			15
Oenanthe leucopyga	67						9	16		3	6
Monticola solitarius	47	11	3	4	5	3	22	3		8	1
Turdus philomelos			115	55	30	75	30	22	29		66
Turdus iliacus		31	104	29	3	82	16	4	13	21	11
Turdus viscivorus	42	11	24	13	10	9	20	6	14	14	1
Turdus pilaris	57		99	31		42	37	11	21	13	6
Turdus merula			143	43	15	77	77	16	34	30	56
Turdus torquatus	69	16	64	15	2	8	23	1	5	5	31
Ficedula hypoleuca		29			34	65	26	2			26
Ficedula albicollis	18	9	4	9		28	11	4		12	17
Lanius isabellinus	67	2	7	2	2	5	2	3		7	8
Lanius senator	42	12	5	6	37	13	17	7		4	41
Lanius e. excubitor	50	10	60	26	1	22	57			2	
Lanius e. aucheri	37	16		12	4	13	20	16			31

Supplementary Table 2. List of data sources for each study species.

		r^2_{model}	df	logLik	GMTA Coefficient	Sex$_{(male)}$ Coefficient	GMTA : Sex$_{(male)}$ Coefficient	AICc	ΔAICc	Akaike Weight
Oenanthe oenanthe	~ GMTA	0.05	3	-1429.80	89.52 ± 35.04	*NA*	*NA*	2865.60	0.00	0.88
	Null Model	0.00	2	-1432.86	*NA*	*NA*	*NA*	2869.70	4.10	0.12
Oenanthe deserti	~ GMTA : Sex$_{(male)}$	0.20	5	-957.77	344.77 ± 87.24	121.02 ± 39.54	-228.81 ± 96.12	1926.00	0.00	0.88
	~ GMTA + Sex$_{(male)}$	0.17	4	-960.83	164.85 ± 37.49	162.37 ± 34.46	*NA*	1930.00	4.00	0.12
	~ GMTA	0.10	3	-970.95	172.71 ± 39.30	*NA*	*NA*	1948.10	22.10	0.00
	Null Model	0.00	2	-980.60	*NA*	*NA*	*NA*	1965.30	39.30	0.00
	Averaged models (ΔAICc < 2)				344.77 ± 87.24	121.02 ± 39.54	-228.81 ± 96.12			
Oenanthe hispanica	~ GMTA + Sex$_{(male)}$	0.05	4	-1395.66	41.52 ± 17.47	-32.59 ± 18.07	*NA*	2799.50	0.00	0.52
	~ GMTA	0.03	3	-1397.32	46.03 ± 17.37	*NA*	*NA*	2800.70	1.20	0.28
	~ GMTA : Sex$_{(male)}$	0.05	5	-1395.66	42.07 ± 34.33	-32.69 ± 18.89	-0.74 ± 39.90	2801.60	2.10	0.18
	Null Model	0.00	2	-1400.84	*NA*	*NA*	*NA*	2805.70	6.20	0.02
	Averaged models (ΔAICc < 2)				43.09 ± 17.56	-32.59 ± 18.07	*NA*			
Oenanthe pleschanka	~ GMTA : Sex$_{(male)}$	0.09	5	-1274.88	152.32 ± 48.83	56.65 ± 28.28	-142.62 ± 58.25	2560.10	0.00	0.86
	~ GMTA + Sex$_{(male)}$	0.05	4	-1277.83	58.27 ± 26.95	80.99 ± 26.29	*NA*	2563.90	3.80	0.13
	~ GMTA	0.02	3	-1282.05	55.12 ± 27.52	*NA*	*NA*	2570.20	10.10	0.01
	Null Model	0.00	2	-1284.00	*NA*	*NA*	*NA*	2572.10	12.00	0.00
	Averaged models (ΔAICc < 2)				152.32 ± 48.83	56.65 ± 28.28	-142.62 ± 58.25			
Oenanthe lugens	~ GMTA	0.08	3	-660.97	96.38 ± 34.80	*NA*	*NA*	1328.20	0.00	0.94
	Null Model	0.00	2	-664.85	*NA*	*NA*	*NA*	1333.80	5.60	0.06
Oenanthe leucopyga	~ GMTA	0.07	3	-709.12	156.53 ± 65.75	*NA*	*NA*	1424.50	0.00	0.87
	Null Model	0.00	2	-712.06	*NA*	*NA*	*NA*	1428.20	3.70	0.13
Monticola solitarius	~ GMTA : Sex$_{(male)}$	0.19	5	-740.83	304.63 ± 115.40	134.52 ± 63.67	-220.81 ± 143.35	1492.30	0.00	0.56
	~ GMTA + Sex$_{(male)}$	0.17	4	-742.18	167.78 ± 69.14	190.61 ± 51.58	-	1492.70	0.40	0.44
	~ GMTA	0.08	3	-749.26	202.29 ± 72.54	-	-	1504.80	12.50	0.00
	Null Model	0.00	2	-753.75	*NA*	*NA*	*NA*	1511.60	19.30	0.00
	Averaged models (ΔAICc < 2)				244.49 ± 119.08	159.17 ± 64.93	-220.81 ± 143.35			
Turdus philomelos	~ GMTA	0.04	3	-2663.46	61.23 ± 14.31	*NA*	*NA*	5333.00	0.00	1.00
	Null Model	0.00	2	-2672.48	*NA*	*NA*	*NA*	5349.00	16.00	0.00
Turdus iliacus	~ GMTA	0.06	3	-1965.94	74.55 ± 17.21	*NA*	*NA*	3938.00	0.00	1.00
	Null Model	0.00	2	-1975.24	*NA*	*NA*	*NA*	3954.50	16.50	0.00
Turdus viscivorus	*Null Model*	0.00	2	-1147.47	*NA*	*NA*	*NA*	2299.40	0.00	0.70
	~ GMTA	0.00	3	-1147.66	-28.62 ± 45.94	*NA*	*NA*	2301.10	1.70	0.30
Turdus pilaris	~ GMTA	0.07	3	-2096.34	128.84 ± 27.55	*NA*	*NA*	4198.70	0.00	1.00
	Null Model	0.00	2	-2106.98	*NA*	*NA*	*NA*	4218.00	19.30	0.00
Turdus merula	~ GMTA : Sex$_{(male)}$	0.03	5	-4110.21	1979.00 ± 374.70	-226.40 ± 191.90	-942.3 ± 448.40	8230.50	0.00	1.00
	~ GMTA	0.02	3	-4119.30	1458.29 ± 222.19	*NA*	*NA*	8244.60	14.10	0.00
	~ GMTA + Sex$_{(male)}$	0.02	4	-4119.20	1452.11 ± 222.17	-43.84 ± 184.29	*NA*	8246.50	16.00	0.00
	Null Model	0.00	2	-4211.19	*NA*	*NA*	*NA*	8426.40	195.90	0.00
	Averaged models (ΔAICc < 2)				1979.00 ± 374.70	-226.40 ± 191.90	-942.3 ± 448.40			
Turdus torquatus	~ GMTA : Sex$_{(male)}$	0.03	5	-1579.22	107.44 ± 39.81	-9.65 ± 25.90	-137.96 ± 57.82	3168.70	0.00	0.47
	Null Model	0.00	2	-1583.01	*NA*	*NA*	*NA*	3170.10	1.40	0.23
	~ GMTA	0.01	3	-1582.17	38.63 ± 28.84	*NA*	*NA*	3170.40	1.70	0.20
	~ GMTA + Sex$_{(male)}$	0.01	4	-1581.82	43.23 ± 29.25	19.95 ± 23.07	*NA*	3171.80	3.10	0.10
	Averaged models (ΔAICc < 2)				87.24 ± 48.43	-9.65 ± 25.90	-137.96 ± 57.82			
Ficedula hypoleuca	*Null Model*	0.00	2	-1210.40	*NA*	*NA*	*NA*	2424.90	0.00	0.53
	~ GMTA	0.00	3	-1210.23	-17.77 ± 31.27	*NA*	*NA*	2426.60	1.70	0.22
	~ GMTA + Sex$_{(male)}$	0.01	4	-1209.62	-15.99 ± 31.24	-33.78 ± 32.08	*NA*	2427.50	2.60	0.14
	~ GMTA : Sex$_{(male)}$	0.01	5	-1208.86	-76.08 ± 55.04	-39.06 ± 32.48	83.10 ± 66.84	2428.10	3.20	0.11
	Averaged models (ΔAICc < 2)				-17.77 ± 31.27	*NA*	*NA*			
Ficedula albicollis	~ GMTA : Sex$_{(male)}$	0.16	5	-843.05	572.29 ± 121.11	345.11 ± 95.88	-427.26 ± 145.98	1696.70	0.00	0.91
	~ GMTA + Sex$_{(male)}$	0.12	4	-846.50	273.05 ± 69.58	341.37 ± 99.08	*NA*	1701.40	4.70	0.09
	~ GMTA	0.10	3	-850.37	229.64 ± 71.87	*NA*	*NA*	1707.00	10.30	0.00
	Null Model	0.00	2	-854.60	*NA*	*NA*	*NA*	1713.30	16.60	0.00
	Averaged models (ΔAICc < 2)				572.29 ± 121.11	345.11 ± 95.88	-427.26 ± 145.98			
Lanius isabellinus	~ GMTA	0.04	3	-874.14	450.00 ± 223.50	*NA*	*NA*	1754.50	0.00	0.56
	~ GMTA + Sex$_{(male)}$	0.04	4	-874.14	444.73 ± 230.24	20.96 ± 207.25	*NA*	1756.70	2.20	0.19
	Null Model	0.00	2	-876.28	*NA*	*NA*	*NA*	1756.70	2.20	0.19
	~ GMTA : Sex$_{(male)}$	0.04	5	-874.14	430.98 ± 381.24	25.14 ± 227.84	21.54 ± 479.69	1758.90	4.40	0.06
	Averaged models (ΔAICc < 2)				450.00 ± 223.50	*NA*	*NA*			
Lanius senator	~ GMTA : Sex$_{(male)}$	0.21	5	-1441.16	445.48 ± 156.49	175.87 ± 96.18	309.60 ± 204.68	2892.60	0.00	0.41
	~ GMTA + Sex$_{(male)}$	0.20	4	-1442.35	628.31 ± 101.25	164.59 ± 96.18	*NA*	2892.90	0.30	0.36
	~ GMTA	0.18	3	-1443.84	628.38 ± 101.99	*NA*	*NA*	2893.80	1.20	0.23
	Null Model	0.00	2	-1462.13	*NA*	*NA*	*NA*	2928.30	35.70	0.00
	Averaged models (ΔAICc < 2)				553.24 ± 155.65	170.60 ± 96.34	-309.60 ± 204.68			
Lanius e. excubitor	~ GMTA	0.02	3	-1590.24	118.21 ± 67.95	*NA*	*NA*	3186.60	0.00	0.81
	Null Model	0.00	2	-1592.73	*NA*	*NA*	*NA*	3189.50	2.90	0.19
Lanius e. aucheri	~ GMTA	0.11	3	-1314.40	960.20 ± 256.40	*NA*	*NA*	2635.00	0.00	0.99
	Null Model	0.00	2	-1320.15	*NA*	*NA*	*NA*	2644.40	9.40	0.01

Supplementary Table 3. The effect of GMTA: list of statistical models, statistics and Akaike Information Criterion (AICc).

		r^2_{model}	df	logLik	Year Coefficient	Sex$_{(male)}$ Coefficient	Year : Sex$_{(male)}$ Coefficient	AICc	ΔAICc	Akaike Weight
Oenanthe oenanthe	~ Year	0.03	3	-1430.83	0.67 ± 0.31	*NA*	*NA*	2867.80	0.00	0.73
	Null Model	0.00	2	-1432.86	*NA*	*NA*	*NA*	2869.80	2.00	0.27
Oenanthe deserti	~ Year : Sex$_{(male)}$	0.21	5	-956.99	3.25 ± 0.88	3437.00 ± 1876.85	-1.69 ± 0.97	1924.40	0.00	0.65
	~ Year + Sex$_{(male)}$	0.19	4	-958.69	1.92 ± 0.38	167.42 ± 33.79	*NA*	1925.70	1.30	0.35
	~ Year	0.12	3	-969.69	1.93 ± 0.40	*NA*	*NA*	1945.60	21.20	0.00
	Null Model	0.00	2	-980.60	*NA*	*NA*	*NA*	1965.30	40.90	0.00
	Averaged models (ΔAICc < 2)				2.78 ± 0.98	2293.62 ± 2173.06	-1.69 ± 0.97			
Oenanthe hispanica	~ Year + Sex$_{(male)}$	0.04	4	-1396.23	0.37 ± 0.18	-31.85 ± 18.22	*NA*	2800.60	0.00	0.49
	~ Year	0.03	3	-1397.79	0.42 ± 0.17	*NA*	*NA*	2801.70	1.10	0.29
	~ Year : Sex$_{(male)}$	0.04	5	-1396.16	0.47 ± 0.34	242.44 ± 770.55	-0.14 ± 0.40	2802.60	2.00	0.18
	Null Model	0.00	2	-1400.84	*NA*	*NA*	*NA*	2805.70	5.10	0.04
	Averaged models (ΔAICc < 2)				0.41 ± 0.22	43.00 ± 420.95	-0.14 ± 0.40			
Oenanthe pleschanka	~ Year : Sex$_{(male)}$	0.09	5	-1274.69	1.78 ± 0.56	3169.84 ± 1279.05	-1.61 ± 0.67	2559.70	0.00	0.85
	~ Year + Sex$_{(male)}$	0.06	4	-1277.57	0.71 ± 0.31	78.16 ± 26.26	*NA*	2563.30	3.60	0.14
	~ Year	0.02	3	-1281.53	0.71 ± 0.31	*NA*	*NA*	2569.20	9.50	0.01
	Null Model	0.00	2	-1284.00	*NA*	*NA*	*NA*	2572.10	12.40	0.00
	Averaged models (ΔAICc < 2)				1.78 ± 0.56	3169.84 ± 1279.05	-1.61 ± 0.67			
Oenanthe lugens	~ Year	0.08	3	-660.46	1.01 ± 0.33	*NA*	*NA*	1327.20	0.00	0.97
	Null Model	0.00	2	-664.85	*NA*	*NA*	*NA*	1333.80	6.60	0.03
Oenanthe leucopyga	~ Year	0.07	3	-709.49	1.38 ± 0.61	*NA*	*NA*	1425.20	0.00	0.82
	Null Model	0.00	2	-712.06	*NA*	*NA*	*NA*	1428.20	3.00	0.18
Monticola solitarius	~ Year : Sex$_{(male)}$	0.19	5	-741.68	2.52 ± 1.01	3799.51 ± 2488.98	-1.88 ± 1.30	1494.00	0.00	0.52
	~ Year + Sex$_{(male)}$	0.17	4	-742.85	1.41 ± 0.64	199.84 ± 51.16	-	1494.10	0.10	0.48
	~ Year	0.08	3	-750.59	1.59 ± 0.67	-	-	1507.40	13.40	0.00
	Null Model	0.00	2	-753.75	*NA*	*NA*	*NA*	1511.60	17.60	0.00
	Averaged models (ΔAICc < 2)				1.98 ± 1.01	2059.04 ± 2537.09	-1.88 ± 1.30			
Turdus philomelos	~ Year	0.04	3	-2661.93	0.72 ± 0.15	*NA*	*NA*	5329.90	0.00	1.00
	Null Model	0.00	2	-2672.48	*NA*	*NA*	*NA*	5349.00	19.10	0.00
Turdus iliacus	~ Year	0.06	3	-1966.67	0.76 ± 0.18	*NA*	*NA*	3939.40	0.00	1.00
	Null Model	0.00	2	-1975.24	*NA*	*NA*	*NA*	3954.50	15.10	0.00
Turdus viscivorus	*Null Model*	0.00	2	-1147.47	*NA*	*NA*	*NA*	2299.40	0.00	0.70
	~ Year	0.00	3	-1147.35	-0.40 ± 0.51	*NA*	*NA*	2300.80	1.40	0.30
Turdus pilaris	~ Year	0.10	3	-2091.32	1.54 ± 0.27	*NA*	*NA*	4188.70	0.00	1.00
	Null Model	0.00	2	-2106.98	*NA*	*NA*	*NA*	4218.00	29.30	0.00
Turdus merula	~ Year : Sex$_{(male)}$	0.03	5	-4130.51	20.52 ± 4.35	18112.71 ± 9890.12	-9.44 ± 5.13	8271.10	0.00	1.00
	~ Year	0.02	3	-4138.98	14.33 ± 2.43	*NA*	*NA*	8284.00	12.90	0.00
	~ Year + Sex$_{(male)}$	0.02	4	-4138.59	14.11 ± 2.43	-98.21 ± 212.31	*NA*	8285.30	14.20	0.00
	Null Model	0.00	2	-4211.19	*NA*	*NA*	*NA*	8426.40	155.30	0.00
	Averaged models (ΔAICc < 2)				20.52 ± 4.35	18112.71 ± 9890.12	-9.44 ± 5.13			
Turdus torquatus	~ Year : Sex$_{(male)}$	0.03	5	-1579.54	1.01 ± 0.40	2668.93 ± 1168.75	-1.38 ± 0.61	3169.30	0.00	0.40
	Null Model	0.00	2	-1583.01	*NA*	*NA*	*NA*	3170.10	0.80	0.28
	~ Year	0.01	3	-1582.26	0.38 ± 0.30	*NA*	*NA*	3170.60	1.30	0.21
	~ Year + Sex$_{(male)}$	0.01	4	-1581.93	0.42 ± 0.30	19.40 ± 23.08	*NA*	3172.00	2.70	0.11
	Averaged models (ΔAICc < 2)				0.80 ± 0.48	2668.93 ± 1168.75	-1.38 ± 0.61			
Ficedula hypoleuca	*Null Model*	0.00	2	-1210.40	*NA*	*NA*	*NA*	2424.90	0.00	0.58
	~ Year	0.00	3	-1210.35	-0.10 ± 0.33	*NA*	*NA*	2426.80	1.90	0.22
	~ Year + Sex$_{(male)}$	0.01	4	-1209.73	-0.09 ± 0.33	-34.20 ± 32.09	*NA*	2427.70	2.80	0.14
	~ Year : Sex$_{(male)}$	0.01	5	-1209.65	-0.26 ± 0.55	-544.08 ± 1350.08	0.26 ± 0.69	2429.60	4.70	0.06
	Averaged models (ΔAICc < 2)				-0.10 ± 0.33	-34.20 ± 32.09	*NA*			
Ficedula albicollis	~ Year : Sex$_{(male)}$	0.15	5	-841.02	6.89 ± 1.16	10953.80 ± 2876.14	-5.50 ± 1.50	1692.60	0.00	0.98
	~ Year + Sex$_{(male)}$	0.09	4	-846.15	3.40 ± 0.79	332.24 ± 97.60	*NA*	1700.70	8.10	0.02
	~ Year	0.09	3	-849.96	2.98 ± 0.83	*NA*	*NA*	1706.10	13.50	0.00
	Null Model	0.00	2	-854.60	*NA*	*NA*	*NA*	1713.30	20.70	0.00
	Averaged models (ΔAICc < 2)				6.89 ± 1.16	10953.80 ± 2876.14	-5.50 ± 1.50			
Lanius isabellinus	~ Year	0.06	3	-872.87	5.84 ± 2.26	*NA*	*NA*	1752.00	0.00	0.65
	~ Year + Sex$_{(male)}$	0.06	4	-872.87	5.86 ± 2.33	-5.11 ± 205.09	*NA*	1754.10	2.10	0.22
	~ Year : Sex$_{(male)}$	0.06	5	-872.86	6.14 ± 3.53	984.39 ± 9072.89	-0.51 ± 4.72	1756.30	4.30	0.07
	Null Model	0.00	2	-876.28	*NA*	*NA*	*NA*	1756.70	4.70	0.06
	Averaged models (ΔAICc < 2)				5.84 ± 2.26	*NA*	*NA*			
Lanius senator	~ Year + Sex$_{(male)}$	0.18	4	-1443.81	6.40 ± 1.06	158.37 ± 96.92	*NA*	2895.80	0.00	0.44
	~ Year	0.16	3	-1445.17	6.40 ± 1.07	*NA*	*NA*	2896.50	0.70	0.32
	~ Year : Sex$_{(male)}$	0.18	5	-1443.34	4.98 ± 1.83	-3968.01 ± 4367.40	2.13 ± 2.25	2897.00	1.20	0.24
	Null Model	0.00	2	-1462.13	*NA*	*NA*	*NA*	2928.30	32.50	0.00
	Averaged models (ΔAICc < 2)				6.06 ± 1.43	-1313.12 ± 3273.32	2.13 ± 2.25			
Lanius e. excubitor	~ Year	0.02	3	-1589.89	1.34 ± 0.68	*NA*	*NA*	3185.90	0.00	0.86
	Null Model	0.00	2	-1592.73	*NA*	*NA*	*NA*	3189.50	3.60	0.14
Lanius e. aucheri	~ Year	0.11	3	-1315.12	9.44 ± 2.69	*NA*	*NA*	2636.40	0.00	0.98
	Null Model	0.00	2	-1320.15	*NA*	*NA*	*NA*	2644.40	8.00	0.02

Supplementary Table 4. The effect of year: list of statistical models, statistics and Akaike Information Criterion (AICc).

Model (PGLS)	λ	r^2_{model}	df	logLik	Migration distance Coefficient	Moult strategy Coefficient	Migration distance : Molt strategy Coefficient	AICc	ΔAICc	Akaike Weight
Null Model	0.03	0.00	1	-139.25	*NA*	*NA*	*NA*	280.70	0.00	0.39
~ Migration distance	0.00	0.09	2	-138.36	-0.05 ± 0.04	*NA*	*NA*	281.50	0.80	0.27
~ Migration distance + Moult strategy	0.00	0.18	3	-137.36	-0.09 ± 0.05	325.51 ± 244.24	*NA*	282.30	1.60	0.18
~ Molt strategy	0.00	0.01	2	-139.18	*NA*	80.57 ± 218.56	*NA*	283.10	2.40	0.12
~ Migration distance * Moult strategy	0.00	0.18	4	-137.33	-0.08 ± 0.05	448.65 ± 652.22	-0.03 ± 0.14	285.50	4.80	0.04
Averaged models (ΔAICc < 2)					-0.07 ± 0.05	325.51 ± 244.24	*NA*			

Supplementary Table 5. Results of analyses of the effects of migration distance (km) and moult strategy (pre- or post-migration moult) on the response of each species to Global Mean Temperature Anomalies (GMTA; slope).

Mean area of moulted feathers (sample size)		*w*	*P*
Lanius* (*excubitor*) *excubitor	***Lanius* (*excubitor*) *aucheri***		
1073.85 mm^2 (228)	8636.55 mm^2 (149)	33968	< 0.001

Supplementary Table 6. The separation of *Lanius excubitor* into two subspecies groups, *Lanius* (*excubitor*) *excubitor* (Great Grey Shrike) and *Lanius* (*excubitor*) *aucheri* (Southern Grey Shrike), is supported by a significant difference in their moult extent (Mann-Whitney U-test).

Species	Feather tract area (mm^2) ± standard deviation (sample size)																					
	LC	MC	GC	CC	AL1	AL2	AL3	PC	T9	T8	T7	S	P1	P2	P3	P4	P5	P6	P7	P8	P9	P10	R
Oenanthe oenanthe	346.2 ± 24.5 (11)	180.5 ± 21.6 (11)	867.5 ± 83.3 (11)						56.2 ± 15.3 (11)	133.7 ± 19.8 (11)													
Oenanthe deserti	356.9 ± 40.5 (4)	184.2 ± 32.5 (4)	866.3 ± 13.0 (4)	39.6 ± 6.9 (4)	21.2 ± 8.0 (4)	44.1 ± 3.9 (4)	41.4 ± 8.5 (4)		55.0 ± 15.8 (4)	115.3 ± 22.6 (4)	125.7 ± 18.6 (4)												1162.1 ± 142.7 (6)
Oenanthe hispanica	303.0 ± 36.1 (10)	160.8 ± 25.9 (10)	658.3 ± 93.9 (10)	32.3 ± 6.4 (10)	16.9 ± 5.6 (10)	41.9 ± 6.1 (10)			49.0 ± 7.7 (10)														
Oenanthe pleschanka	370.4 ± 94.3 (3)	182.9 ± 27.3 (3)	841.5 ± 158.1 (3)	40.3 ± 5.4 (3)	17.7 ± 5.6 (3)				52.9 ± 4.3 (3)	122.4 ± 39.8 (3)	161.3 ± 24.3 (3)												
Oenanthe lugens	311.1 ± 39.4 (7)	162.2 ± 22.4 (7)	737.3 ± 70.1 (7)	32.3 ± 8.4 (7)	22.1 ± 6.1 (7)	48.8 ± 7.5 (7)	48.0 ± 8.8 (7)		66.2 ± 15.8 (7)	112.3 ± 23.1 (7)	128.0 ± 32.8 (7)												1186.0 ± 151.3 (6)
Oenanthe leucopyga	374.5 ± 70.0 (4)	223.5 ± 37.7 (4)	912.5 ± 128.5 (4)	41.7 ± 4.4 (4)	27.8 ± 8.2 (4)	58.6 ± 7.8 (4)	53.6 ± 9.3 (4)		80.2 ± 20.0 (4)	140.3 ± 17.2 (4)	169.6 ± 54.7 (4)												1497.7 ± 68.9 (4)
Monticola solitarius	563.9 ± 50.2 (5)	345.9 ± 71.3 (5)	1267.5 ± 132.4 (5)	86.7 ± 22.0 (5)					73.2 ± 10.4 (5)	172.4 ± 13.3 (5)													2503.0 ± 490.3 (5)
Turdus philomelos	495.5 ± 67.6 (4)	372.3 ± 28.0 (4)	1180.1 ± 146.0 (4)						82.8 ± 20.8 (4)	217.1 ± 43.3 (4)													
Turdus iliacus	552.9 ± 125.3 (4)	332.8 ± 53.2 (4)	1159.4 ± 126.4 (4)																				
Turdus viscivorus	736.4 ± 143.3 (4)	489.2 ± 67.9 (4)	1645.8 ± 125.6 (4)	60.6 ± 18.4 (4)	52.3 ± 16.0 (4)				127.5 ± 38.3 (4)														
Turdus pilaris	641.4 ± 109.9 (4)	402.8 ± 35.9 (4)	1553.5 ± 200.5 (4)						109.2 ± 41.3 (4)	316.2 ± 83.5 (4)													
Turdus merula	681.3 ± 87.2 (4)	395.9 ± 31.5 (4)	1359.4 ± 71.9 (4)	46.4 ± 13.6 (4)	33.6 ± 5.2 (4)	95.6 ± 14.1 (4)	88.9 ± 23.5 (4)	804.9 ± 79.8 (4)	62.2 ± 38.2 (4)	232.3 ± 17.7 (4)	260.1 ± 68.4 (4)	2892.2 ± 252.8 (4)	410.5 ± 36.2 (4)	544.7 ± 32.4 (4)	535.0 ± 46.8 (4)	624.9 ± 63.4 (4)	705.2 ± 22.5 (4)	670.2 ± 25.1 (4)	619.9 ± 69.9 (4)	590.2 ± 49.8 (4)	416.3 ± 39.4 (4)	42.1 ± 13.1 (4)	3583.2 ± 395.6 (5)
Turdus torquatus	742.7 ± 170.1 (4)	427.0 ± 62.4 (4)	1521.3 ± 115.4 (4)																				
Ficedula hypoleuca	201.8 ± 22.3 (5)	95.2 ± 14.2 (5)	473.0 ± 27.8 (5)	20.1 ± 3.1 (5)	12.3 ± 0.9 (5)				35.7 ± 9.7 (5)	82.6 ± 10.0 (5)	109.9 ± 13.7 (5)	1019.8 ± 56.2 (5)											893.8 ± 116.0 (5)
Ficedula albicollis	215.5 ± 17.1 (10)	111.2 ± 10.7 (10)	457.4 ± 37.4 (10)	26.0 ± 4.0 (10)	11.4 ± 2.8 (10)	29.1 ± 6.5 (10)	41.8 ± 6.3 (10)	314.4 ± 21.7 (10)	34.8 ± 7.3 (10)	81.9 ± 6.9 (10)	105.2 ± 9.5 (10)	993.1 ± 84.8 (10)	182.1 ± 16.5 (10)	214.5 ± 20.8 (10)	215.1 ± 18.1 (10)	231.3 ± 16.9 (10)						26.3 ± 9.2 (10)	864.6 ± 127.2 (5)
Lanius isabellinus	382.1 ± 79.2 (8)	210.1 ± 30.3 (8)	860.7 ± 83.6 (8)	56.4 ± 17.4 (8)	17.6 ± 2.6 (8)	41.0 ± 4.8 (8)	57.7 ± 16.5 (8)	447.8 ± 34.9 (8)	70.8 ± 17.5 (8)	163.2 ± 19.9 (8)	210.4 ± 36.5 (8)	1607.5 ± 106.8 (8)	275.6 ± 44.8 (8)	315.6 ± 21.2 (8)	311.0 ± 11.8 (8)	349.2 ± 19.7 (8)	371.8 ± 27.3 (8)	381.5 ± 32.9 (8)	406.9 ± 45.1 (8)	378.9 ± 23.0 (8)	273.7 ± 29.8 (8)	45.2 ± 13.2 (8)	1813.3 ± 171.9 (4)
Lanius senator	380.2 ± 84.1 (7)	225.9 ± 23.9 (7)	913.9 ± 115.0 (7)	52.9 ± 26.4 (7)	13.7 ± 4.2 (7)	49.0 ± 9.8 (7)	67.6 ± 16.3 (7)	472.7 ± 28.8 (7)	64.0 ± 13.7 (7)	149.5 ± 23.5 (7)	174.7 ± 21.5 (7)	1453.2 ± 185.2 (7)	247.5 ± 50.1 (7)	308.7 ± 26.7 (7)	305.9 ± 46.7 (7)	344.2 ± 29.3 (7)	350.9 ± 42.1 (7)	391.3 ± 38.3 (7)	432.1 ± 25.6 (7)	428.1 ± 24.9 (7)	338.1 ± 16.2 (7)	43.9 ± 10.6 (7)	1876.1 ± 276.3 (5)
Lanius excubitor	477.1 ± 61.7 (6)	316.3 ± 60.6 (6)	1170.5 ± 119.6 (6)	80.2 ± 18.3 (6)	26.3 ± 7.9 (6)	70.1 ± 7.8 (6)	65.9 ± 12.3 (6)	672.0 ± 117.0 (6)	94.8 ± 18.2 (6)	209.0 ± 30.8 (6)	198.6 ± 56.4 (6)	1895.0 ± 142.5 (6)	450.4 ± 77.4 (6)	452.3 ± 89.5 (6)	428.5 ± 68.3 (6)	433.5 ± 71.9 (6)	515.7 ± 32.9 (6)	539.7 ± 35.1 (6)	592.0 ± 57.0 (6)	530.1 ± 60.5 (6)	401.8 ± 19.4 (6)	121.0 ± 22.0 (6)	3209.7 ± 389.7 (5)

Supplementary Table 7. Feather area of moulted feathers in the study species.
